# Achieving Continuity of Care: Facilitators and Barriers in Community Mental Health Teams

**DOI:** 10.1186/1748-5908-6-23

**Published:** 2011-03-18

**Authors:** Ruth Belling, Margaret Whittock, Susan McLaren, Tom Burns, Jocelyn Catty, Ian Rees Jones, Diana Rose, Til Wykes

**Affiliations:** 1 Department of Psychiatry, University of Oxford, Warneford Hospital, Oxford OX3 7JX, UK; 2 Division of Mental Health, St George's, University of London, Jenner Wing, Cranmer Terrace, London SW17 0RE, UK; 3 Institute of Strategic Leadership and Service Improvement, Faculty of Health and Social Care, London South Bank University, 103 Borough Rd, London SE1 0AA, UK; 4 School of Social Sciences, Bangor University, University of Wales, Bangor, Gwynedd LL57 2DG, UK; 5 Department of Psychology, PO Box 77, Institute of Psychiatry, King's College London, De Crespigny Park, London SE5 8AF, UK

## Abstract

**Background:**

The integration of mental health and social services for people diagnosed with severe mental illness (SMI) has been a key aspect of attempts to reform mental health services in the UK and aims to minimise user and carer distress and confusion arising from service discontinuities. Community mental health teams (CMHTs) are a key component of UK policy for integrated service delivery, but implementing this policy has raised considerable organisational challenges. The aim of this study was to identify and explore facilitators and barriers perceived to influence continuity of care by health and social care professionals working in and closely associated with CMHTs.

**Methods:**

This study employed a survey design utilising in-depth, semi-structured interviews with a proportionate, random sample of 113 health and social care professionals and representatives of voluntary organisations. Participants worked in two NHS Mental Health Trusts in greater London within eight adult CMHTs and their associated acute in-patient wards, six local general practices, and two voluntary organisations.

**Results:**

Team leadership, decision making, and experiences of teamwork support were facilitators for cross boundary and team continuity; face-to-face communication between teams, managers, general practitioners, and the voluntary sector were facilitators for information continuity. Relational, personal, and longitudinal continuity were facilitated in some local areas by workforce stability. Barriers for cross boundary and team continuity were specific leadership styles and models of decision making, blurred professional role boundaries, generic working, and lack of training for role development. Barriers for relational, personal, and longitudinal continuity were created by inadequate staffing levels, high caseloads, and administrative duties that could limit time spent with users. Incompatibility of information technology systems hindered information continuity. Flexible continuity was challenged by the increasingly complex needs of service users.

**Conclusions:**

Substantive challenges exist in harnessing the benefits of integrated CMHT working to deliver continuity of care. Team support should be prioritised in terms of IT provision linked to a review of current models of administrative support. Investment in education and training for role development, leadership, workforce retention, and skills to meet service users' complex needs are recommended.

## Background

The integration of mental health and social services for people diagnosed with severe mental illness (SMI) has been a key aspect of attempts to reform mental health services in the UK [1], with the aims of minimising user and carer distress and confusion arising from service discontinuities and addressing major issues such as service fragmentation [2], poor interdisciplinary communication, co-ordination [3], and decision making [4]. Community Mental Health Teams (CMHTs) are a key component of UK policy for integrated service delivery [5], providing continuity of care by harnessing the mix of professional skills drawn from medicine, psychology, social work, nursing, and occupational therapy into multidisciplinary teams, each expected to have clear leadership, use one set of notes, and achieve geographical co-location of team members.

Implementing policy reform has raised a number of challenges for delivering on continuity of care from an organisational perspective. Continuity of care is a multifaceted concept that can be defined and operationalised in different ways according to a recent scoping study [6]. Challenges for continuity encompass the implementation of systems for effective information transfer within and across organisational boundaries, together with the provision of consistent information to users and carers (information continuity); the effective co-ordination of services by teams, external agencies, users, and carers (cross boundary/team continuity); the development of flexible care plans linked to effective monitoring (flexible continuity); the deployment of professional staff to remove disjointed episodes of service delivery (longitudinal continuity); the designation and accountability of one or more professional staff to foster therapeutic relationships and exert a positive impact on care outcomes (relational, personal, and therapeutic continuity); and the development of systems and processes to provide care adequate to meet needs over time (long-term continuity).

In the early stages following policy implementation, communication, co-ordination and decision-making difficulties, concerns over loss of professional identity [7,8], limited resources, lack of time [9], bureaucracy [10], and leadership [11] in establishing effective CMHTs were reported. However, early studies focused on isolated aspects of team working and did not explore organisational barriers and facilitators that can impact on continuity of care from a wide range of professional perspectives. This qualitative investigation explored these factors in depth in two NHS mental health Trusts.

### Wider context: The ECHO study

This qualitative work formed a key part of the organisational strand of a multi-phase study 'Experiences of Continuity of Care and Health and Social Care Outcomes: The (ECHO) Study' funded by the National Co-ordinating Centre Service and Delivery Organisation (NCCSDO). In addition to the organisational strand, selected findings of which are the subject of this paper, partner ECHO strands included a developmental phase focused on the generation of user and carer measures of continuity of care; the main phase investigating health and social care outcomes in service users with psychotic and non-psychotic disorders together with carer experiences of continuity, caring, and impact on carer psychological well-being. A qualitative strand, focused on the experiences and views of service users purposively sampled, was based on findings of the main phase. Findings of the wider ECHO study are located in the final NCCSDO report [12] and recent publications, also referred to in the discussion section to this paper [13,14].

### Ethics

Prior to commencing the study, ethical approval was gained from two local research ethics committees (LREC) associated with each Trust. Informed consent to take part in interviews was obtained from participants. Principles of confidentiality and anonymity together with the requirements of the Data Protection Act have been applied in the conduct, reporting, and storage of data arising from this study in accordance with LREC requirements.

## Methods

The objective was to identify and explore facilitators and barriers perceived to influence continuity of care by health and social care professionals working in adult multidisciplinary CMHTs and associated acute wards, general practices, and representatives of voluntary organisations. A survey was conducted in two NHS mental health Trusts in greater London. Together, the Trusts delivered services across nine London boroughs. Multidisciplinary CMHTs in both Trusts had implemented the care programme approach, in which each patient is managed by a key worker, who is a health professional, in association with a consultant psychiatrist. The survey comprised a structured questionnaire, results reported separately [12], followed by semi-structured, in-depth interviews reported in this paper. An interview schedule was developed, based on both questionnaire findings and six pilot fieldwork interviews, to explore health and social care professionals' experiences of integrated working in relation to delivering continuity of care.

Interviews were conducted in 2005 and 2006, and analysed and reported in 2006 and 2007. The final report of the ECHO Study was finalised in December 2007. Following ethical approval and as part of essential preparatory fieldwork, written information about the study objectives and participation was circulated to all potential CMHT and non-CMHT participants, and presentations were made to key stakeholders in each Trust. Interviews were conducted with a randomly selected proportionate sample of health and social care professionals (n = 113), including team and line managers, working in eight CMHTs (four per Trust), together with their closely associated acute in-patient wards, GPs representing six local GP practices and representatives of two voluntary organisations working within the same geographical location. Refusal rate in the population sampled was 31% and comparable to that of the questionnaire. Sample descriptors according to professional group and operational/management status by Trust are summarized in Tables 1 and 2.

**Table 1:** Professional groups by NHS Trust

N (% overall total) Profession	Trust 1 (N = 52)	Trust 2 (N = 61)	Total (N = 113)
Psychiatrist	4 (3.5%)	2 (1.7%)	6 (5.3%)

Psychologist	3 (2.6%)	3 (2.6%)	6 (5.3%)

Social Worker	13 (11.5%)	10 (8.8%)	23 (20.3%)

Nurse	23 (20.3%)	29 (25.6%)	52 (46.0%)

Occupational Therapist	2 (1.7%)	6 (5.2%)	8 (7.0%)

General Practitioners	3 (2.6%)	3 (2.6%)	6 (5.3%)

Voluntary Sector Workers	3 (2.6%)	5 (5.2%)	8 (7.0%)

Support Workers	1 (0.8%)	1 (0.8%)	2 (1.7%)

Non Health and Social Care Professionals^1^	0 (0%)	2 (1.7%)	2 (1.7%)

^1^Managers without professional health/social care qualifications whose roles impacted service delivery

**Table 2:** Managerial/Operational Status by NHS Trust

N (% of overall total)	Trust 1 (N = 52)	Trust 2 (N = 61)	Total (N = 113)
Managers: CMHTs	15 (13.3%)	20(17.7%)	35 (31.0%)

Managers: Non CMHTs	6 (5.3%)	8 (7.1%)	14 (12.4%)

Operational Staff: CMHTs	23 (20.3%)	22 (19.5%)	45 (39.8%)

Operational Staff: Non CMHTs	8 (7.1%)	11 (9.7%)	19 (16.8%)

MW and RB carried out interviews (duration 45 to 60 minutes). These were audiotaped, transcribed verbatim, checked for accuracy, and then entered into QSR NU*DIST v.6 software to assist the data analysis. Data were systematically coded, categorised, and analysed using 'framework analysis' [15], where data are categorised according to a structured framework reflecting the research aim as embodied within the interview schedule, participants' emerging issues and recurrent themes. Framework analysis has five stages: familiarisation with data; identifying a thematic framework; indexing, labelling, and sorting data; creation of thematic charts; and mapping and interpretation.

## Results

This paper presents the following themes identified from the framework analysis: teamwork; workforce stability; communications; leadership and decision making models; professional role boundaries; generic working; support for training and role development; information systems; workforce levels/workloads; and service users' needs. Findings from themes defining continuity of care and change management form the subject of a separate paper. Table 3 shows the three themes and four subthemes perceived as facilitators to continuity of care, while the seven themes and nine subthemes perceived as barriers to continuity of care are shown in Table 4. Themes are organised and discussed below within the context of the different definitions of continuity of care identified in the scoping study [6]. Illustrations of each of the facilitator and barrier themes are presented in Additional file 1: Table S1 and Additional file 2: Table S2, respectively, and signposted within the narrative by table, theme, and subtheme where relevant.

**Table 3:** Facilitators to continuity of care

Theme	Sub-themes
Teamwork	Teamwork support Team leadership/decision-making

Workforce stability	(None)

Communications	Team and managers Voluntary sector and GPs

**Table 4:** Barriers to continuity of care

Theme	Sub-themes
Leadership and decision making models	(None)

Professional role boundaries	(None)

Generic working	(None)

Support for training and role development	(None)

Information systems	Incompatibility IT provision

Workforce levels/workloads	Pressures on staffing levels Recruitment, retention, staff sickness Caseloads/case management Administrative loads Impact on communication

Service users' needs	Complexity of needs Accommodation

### Cross boundary and team continuity

Positive experiences of teamwork support, leadership and decision making (Additional file 1: Table S1, theme: teamwork) were identified as facilitators to continuity. Support of team members was important in creating a positive working environment marked by shared discussion, equitable workloads, and effective leadership. New models of team leadership had emerged (Trust two), which were seen by some to be more empowering and democratic in terms of impact on decision making, with leaders drawn from a range of professional groups and consultant psychiatrists retaining clinical responsibilities. However, not all experiences had been positive (Additional file 2: Table S2, theme: team leadership and decision-making models) where medical models of decision making were perceived to dominate and team leaders underperformed, creating dilemmas for the consultant psychiatrist in maintaining the service.

Many participants expressed anxiety at the perceived erosion of their professional roles and identities due to generic and cross-boundary working (Additional file 2: Table S2, themes: professional roles and boundaries; generic working). Reservations related to taking on tasks for which participants felt they had no training or experience; for CPNs and social workers, examples included taking on aspects of social care and involvement in monitoring medication effects, respectively. In Trust two, professional boundaries had been maintained through retention of a separate team identity for psychologists outside the formal CMHT service structure.

Although both Trusts provided mandatory and discretionary training, education and continuing professional development opportunities for professionals, lack of preparation for generic working, and lack of training for the acquisition of other skills relevant to role development were perceived negatively in some teams; accessibility of training was also seen as problematic (Additional file 2: Table S2, theme: support for training and role development). In Trust one, team leaders had not been provided with management training for their leadership role.

### Information continuity

Facilitators for information continuity (Additional file 1: Table S1, theme: teamwork, sub-theme: team leadership and decision making) included regular team meetings reinforced by the benefits of geographical co-location that enhanced information exchange; inclusivity in case review meetings involving users, carers, and professionals, set against an organisational background of greater transparency of information; and communication with the voluntary sector and general practice (Additional file 1: Table S1, theme: communication; sub-theme: voluntary sector and general practice). Barriers to information continuity (Additional file 2: Table S2) were the inadequate provision of information technology (IT) resources (Additional file 2: Table S2, theme: information systems; sub-theme: IT provision). Challenges had arisen from the need to combine two entirely separate computerized methods of recordkeeping by health and social services for use by integrated CMHTs. Incompatibilities in existing software packages, difficulties encountered in using new packages, and limited quality and quantity of IT equipment were barriers for recording information and communication. Competition for available computers had led to shifts in working patterns, lengthening the working day for some staff.

### Relational, personal, therapeutic, and longitudinal continuity

Both Trusts operated care programme approaches, allocating case managers to users to foster therapeutic continuity. However, organisational factors (workforce stability, vacancies, turnover, use of temporary staff, workloads) impacted both positively and negatively on both therapeutic and longitudinal continuity. Relational and personal continuity were facilitated by improvements in workforce stability in Trust one, where positive strategies to recruit newly qualified nurses who had trained within the Trust and offer qualified staff a development scheme to enhance professional development had reduced vacancy rates. In Trust two, implementing management strategies to prevent movement of CMHT members within the organisation to fill in gaps in service delivery had supported continuity (Additional file 1: Table S1, theme: workforce stability).

Barriers that threatened both therapeutic and longitudinal continuity (Additional file 2: Table S2, theme: workforce levels and workloads) included inadequate staffing levels, staff absences, and a resulting reliance on temporary agency workers who were not always perceived to be suitable for the required role. Many participants remarked on financial pressures that had resulted in staffing cutbacks, increasing caseloads and caseload management. Voluntary service workers noted a negative impact on time for communication due to time pressures arising from heavy workloads. Some team leaders struggled to subsume caseloads similar to team members alongside their management responsibilities. Increases in caseloads, administration, and paperwork in both Trusts could impact negatively on staff attrition, patient contact time, user discharge, and continuity (Additional file 2: Table S2, theme: workforce levels and workloads; sub-themes: caseloads, case management; administrative loads). In some cases, the underlying problems were lack of computing resources and diversion of scarce secretarial support.

### Flexible and long-term continuity

Participants in both Trusts indicated that the often complex nature of service users' mental health needs could be a barrier to providing continuity of care (Additional file 2: Table S2, theme: service users' needs; sub-theme: complexity of needs). Reasons for this were that the nature of the illness could mean service users might not comply with treatment, needs may change, and services could fail to keep up with these changes. Difficulties were experienced in making and maintaining contact with vulnerable people and a scarcity of accommodation for this changing population, especially those with 'dual diagnosis' drug and alcohol-related behavioural problems. Participants needed to be better prepared for the growing challenges of violence and substance misuse, combined with other mental health problems. Shortages of user accommodation also hindered the ability for services to be flexible, adjusting to the needs of individuals over time (Additional file 2: Table S2, theme: service users' needs; sub-theme: accommodation).

## Discussion

Over the last decade, the delivery of integrated mental health and social services for people diagnosed with SMI has been a central plank of policy reform in the UK [1]. To what extent has the delivery of services by integrated CMHTs addressed original concerns relating to lack of continuity of care, poor communication, co-ordination, and decision making? What are the current facilitators and barriers perceived by health and social care professionals that can impact on continuity of service delivery? Findings from this study should be interpreted in the light of a number of strengths and limitations. Relatively high participation in the interview (and questionnaire) components of this survey, together with the use of random proportionate sampling, assist in attenuating bias. Other strengths are that these findings of the organisational strand have been supported by those of other strands within the ECHO study, enhancing the validity of findings. Limitations that constrain generalisation of the findings arise from the geographical location of the study in Trusts within rural and urban settings in the greater London area, where organisational factors affecting continuity of care in relation to workforce deployment and stability (staff recruitment, retention, and turnover) may differ from other UK settings.

Findings relating to the experiences of health and social care professionals suggest that, while progress has been made, a number of barriers can frustrate and impede multi-disciplinary working in CMHTs, with potential negative impacts on continuity.

A requirement for information to follow the patient so it is available wherever and whenever needed is intrinsic to achieving both information, flexible, and long-term continuity in a patient-centred NHS [10]. Consistency of information provided by health and social care professionals to users, underpinned by the need for professionals to share information related to monitoring observations, assessments, care plans, and discharge/transfer to other care settings is vital [11], and provision of adequate IT systems is fundamental to service delivery. A challenge for information, flexible, and long-term continuity is the high degree of mobility documented for users with a serious mental illness, which can result in loss of contact with service providers and the complexity of interfaces for information transfer within and between acute, primary care, and voluntary sector organisations [16].

Geographical co-location of health and social care professionals within CMHTs, linked with positive management strategies that enhanced face to face communication with users, carers, and professionals from both statutory and voluntary sectors were identified in this study as facilitators for decision making and continuity. However, inadequate provision of IT equipment was a barrier for information, flexible and long-term continuity, due to incompatibility of software systems, use of outdated computer hardware, which in some cases was shared with other professionals, and lack of finance to update provision. These findings reinforce earlier concerns [17] raised at the time of service integration and emphasise current concerns about the time delays which have affected IT programme innovation in the NHS [18], where it is intended that a phased process will address priorities in implementing IT developments over several years [10]. From the perspective of information, flexible and long-term continuity, these findings support the need for CMHT services to be prioritised in terms of IT resources.

Findings of the organisational strand of ECHO relating to informational, personal, and therapeutic continuity both support and are reinforced by selected findings of other study phases of ECHO. Continuity domains rated as very important by service users in the main phase included staff changes, information provision, and communication [13]. Interviews with service users and carers reported within the qualitative strand [14] have revealed good and bad 'depersonalised transitions' marked in some negative cases by poor communication and information provision (notable at discharge and between services/voluntary agencies), together with relational discontinuities emanating from repeated turnover of professional staff, particularly key workers. With regard to staff turnover, service users and carers expressed frustration at the time needed to build up new relationships, continually having to repeat information about their personal circumstances and re-tell their stories.

In relation to cross-boundary and team continuity [19], key findings endorse those of studies [20,21] conducted in the earlier stages of integration in that the majority of professionals in both organisations had positive experiences of working in co-located, integrated, multidisciplinary teams and these facilitated continuity. However, tensions and conflicts over professional identities, role blurring and challenges for working across professional boundaries were illustrated by the co-existence of a separate team of psychologists in one organisation. Generic working, intended from a management perspective to broaden the skills profile of a team and enhance service delivery, was a source of concern, particularly where training for new aspects of roles -- for example, medication management by social workers -- had not been provided, raising questions about quality and safety. These findings support concerns expressed prior to service integration [21-23].

In addition to lack of specific training opportunities and role conflicts, leadership was also identified as a problematic issue by professionals working in some CMHTs. In one Trust, a traditional 'medical model' was common, where a psychiatrist led the team. For some professionals, issues arose about power sharing and decision making where authoritarian styles (negatively perceived) predominated. In the other Trust, teams had been restructured to allow leadership by other professionals, with a move toward a more democratic process of decision making. However, in the latter, poor quality of leadership had been identified by some medical consultants. This could reflect a lack of training for leadership and management, or resistance to the move away from medically dominated hierarchies.

Continuity of care remains a high national priority within the UK. In the context of our findings how can we 'start from here' to ensure a supportive service is delivered for people with enduring or episodic health problems? Tighter national finances and the abolition of PCTs mean it is highly unlikely that the more obvious means of reducing the barriers restricting mental health services' capacity to deliver care continuity, notably through increased resources in staffing, service users' day care and accommodation and computing, will be realised. Workforce levels and facilities for service users remain vital, however and their resource levels must be protected wherever possible. Nevertheless, within the current climate, these findings suggest several areas where continuity of care can realistically be sustained and improved, particularly through service users' needs and priorities, workforce communication, and team leadership.

Times of austerity present an opportunity to refocus on service users' needs and priorities, many of which are highlighted in the wider ECHO study findings [12]. This strand's findings highlight current needs for some form of community day care and accommodation, together with newer, emerging needs for younger people with multiple diagnoses. Though this study was limited to adult mental health, recent research into transition from child and adolescent mental health services [24] suggests stronger links are needed between the two, to reinforce continuity over a person's lifespan, better preparing young people to engage with adult services, and preventing the most vulnerable re-engaging with mainstream services only at crisis points and often at great personal cost to the individual and to already stretched services.

Communication between teams becomes increasingly more important when workforce levels are unstable and information systems often frustrating rather than helpful. Good administrative support can boost communications and team efficiency. The views of administrative staff in CMHTs were not included within this study's sample, but they act as gatekeepers both to staff and information and their contribution could perhaps be further maximised. Similarly, team leadership is a critical component with team leaders fulfilling pivotal roles in maintaining cohesive teams, reducing outside pressures, and creating supportive environments in which staff are able to operate and develop. Yet, in many cases within this study, team leaders had not received any training or development for their crucial roles. Finding ways to support their development should also be prioritised.

Future directions of research indicated by this study include evaluations of team building, leadership, and decision-making training interventions on staff, service user, and organisational outcomes in CMHTs. Can we meet these challenges and priorities for innovation in prioritising service support and continuing research? Only time will tell.

## Conclusions

Policy implementation regarding CMHT integration has raised many practice issues yet to be resolved. Strategies are needed to maximize recruitment and retention of staff and minimize workforce turnover. Services supporting the care of people diagnosed with SMI should be prioritized in terms of IT provision linked to a review of current models of decision making and administrative support. Training should be prioritized in integrated team working and team leadership, role development and competencies within CMHTs, change management, and management of temporary workers. Models of care to meet service users' complex care needs for dual diagnosis should be developed and adequately resourced. A review of accommodation resources to support continuity of care is urgently needed in service settings.

## Competing interests

The authors declare that they have no competing interests.

## Authors' contributions

RB, SM and MW substantially contributed to conception and design of the paper. MW, RB and SM substantially contributed to collection, analysis, and interpretation of data. RB drafted the article. RB and SM revised the article critically for important intellectual content. TB, JC, SM, IRJ, DR, and TW contributed to conception and design of the wider ECHO study. All contributors approved the final version.

## Supplementary Material

Additional file 1 **Facilitators to continuity of care**. Illustrative extracts of themes and sub-themes: facilitators to continuity of care. Click here for file

Additional file 2 **Barriers to continuity of care**. Illustrative extracts of themes and sub-themes: barriers to continuity of care. Click here for file
