# Supplementary material for: Achieving Continuity of Care: Facilitators and Barriers in Community Mental Health Teams
Source: Implement Sci. 2011 Mar 18;6:23. doi: 10.1186/1748-5908-6-23 (PMC3073925; doi:10.1186/1748-5908-6-23)
Supplement: Additional file 1 — Facilitators to continuity of care. Illustrative extracts of themes and sub-themes: facilitators to continuity of care. [file 1748-5908-6-23-S1.DOC]

**Table S1. Facilitators to continuity of care**

**Teamwork:**

- **Experiences of teamwork support**

‘I think there is a tremendous goodwill amongst team members to help out colleagues and generally not to dump work on other people so I think this sense of there being a good team atmosphere here and I think that counts for a lot when you’re under pressure and team members being understanding of other team members and difficulties really.’ (Social Worker, Trust two)

‘I think a good team; I think that makes all the difference. It really is important because I could be doing the same work in a bad team and then you don’t have the support, you know you cant discuss things and bounce ideas off and get that support.’ (CPN Trust one)

‘I feel they all take on the same kind of interests, they don’t object to doing one thing or the other, there aren’t different rules for one lot as far as the clinical workload is concerned.’ (Consultant Psychiatrist, Trust one)

‘Fortunately we have a very good practice manager and we have a very good support team so that work is delegated as appropriate within the practice so that eases the burden on Doctors.’

(GP Trust two)

- **Team leadership and decision making**

**‘**I think the team leader is a good idea, I think that’s going to help pull all the strands together and look at gaps. And it means somebody is actually looking at a vision, because we don’t have enough time to look at the vision.’ (Occupational Therapist, Trust two)

‘I think the new team leader structure has empowered the team leaders. For the first time, some of the other consultant psychiatrists are finding people saying what’s this about; I disagree with this…it’s uncomfortable but it needs to happen. We have increased democracy, increased empowerment and say in how our services are run and delivered and changed.’ (Senior Manager, Trust two)

‘I think it’s almost as democratic as you can get, although people understand that the consultants have clinical responsibility so sometimes someone will have to make a ruling, but it is about as democratic a meeting as you can get.’ (Team Leader, Social Worker, Trust one)

‘We chair, we take it in turns, it varies between six monthly and three monthly, but everybody chairs the meeting, I feel that everybody should have a go. Normally the decisions are made as a team and I can’t even think of an occasion when I over-ruled…..but I take responsibility obviously for decisions made.’ (Consultant Psychiatrist, Trust one)

**Workforce stability**

‘Well actually, we have over the last year improved our retention. Our recruitment has improved and that’s through initiatives that we’ve put in place to bring turnover down.’ (Senior Manager, Nurse, Trust one)

‘We have a lot of staff here who have been here for quite a lot of time which helps continuity of care. People are familiar with relationships between various family members over the years and that makes it easier.’ (Consultant Psychiatrist, Trust one)

‘The main thing is that now staff are not just willy-nilly moved around, that’s stopped, its much more settled and that is so important within the team as a whole.’ (Team Leader, CPN, Trust two)

**Communication:**

- **Teams and managers**

‘I think on the whole we communicate quite well, we have team meetings and we talk about the patients, any particular problems...’ (Occupational Therapist, Trust one)

‘Communication I think is one of the key areas where we have really improved over the last year. People do know much more about the Trust now I think and what’s going on than they did. There seems to be less secrecy.’ (Senior Manager, Social Worker, Trust two)

‘The facilitators are having true multidisciplinary communication and having people together in one place to do that on a regular basis, and I don’t just mean once a week, I mean the stuff that goes on when you share a space. I think that’s a huge plus, because decisions don’t get deferred, they get made.’ (Senior Manager, Occupational Therapist, Trust two)

- **Voluntary sector and general practice**

‘We have good links with most of our GPs which facilitates discussions about referrals for people... We have other people in the team who have good other relationships so our OT has a relationship with MIND and the employment service and that is quite good.’ (Consultant Psychiatrist, Trust one)

‘...we have a three monthly meeting with our GPs and we invite relatives and all the professions concerned as well. Everything is discussed and the care plans reviewed.’ (Team Leader, CPN, Trust one)

‘For me, my main thing is community people because they are the people I need to be in contact with. So the volunteer bureau, the disability employment advisors, the day centres…it’s really important to have a good relationship with the people in the community, because at the end of the day if we don’t, it’s really hard to get people (service users) involved in things.’ (Occupational Therapist, Trust two)
