# Supplementary material for: Achieving Continuity of Care: Facilitators and Barriers in Community Mental Health Teams
Source: Implement Sci. 2011 Mar 18;6:23. doi: 10.1186/1748-5908-6-23 (PMC3073925; doi:10.1186/1748-5908-6-23)
Supplement: Additional file 2 — Barriers to continuity of care. Illustrative extracts of themes and sub-themes: barriers to continuity of care. [file 1748-5908-6-23-S2.DOC]

**Table S2. Barriers to continuity of care**

**Leadership and decision-making models**

‘I think there is a pull towards a more medical model. And I think it is harder for people to maintain their own sense of where they came from. The style of consultant psychiatrists has an effect on that. I’ve been able to observe different consultants at work and the less authoritarian the consultant is, the freer people are to express their own identity, if you like. The doctors having to be the main people, seems to be unnecessary, so people might be assessed a number of times, because they need the medical input first. The services are currently being reviewed, to try to change the way the system works, to become a more psychological model.’ (Psychologist, Trust two)

‘…it obviously raises issues about the quality of… and support to the team leaders because they become crucial. There are other parts of (the geographical area) where my consultant colleagues report that their life is made a misery by poor quality team leaders so they have to sort of carry a service without the nominal responsibility, but still keep the show going. It is a terribly difficult role.’ (Consultant Psychiatrist, Trust two)

**Professional roles and boundaries**

‘I think integration is a good thing but I have reservations whether we should all be doing the same thing. I think as a nurse myself I would be reluctant to give up that arm because…I have trained five years as a nurse and I want to retain my practice as a nurse … I would like to protect the nursing name I suppose.’ (CPN, Trust one)

‘I’m an OT working as a team worker so I need to have a good part of my time doing work that only I can do, otherwise you might as well not have me as an OT, have somebody else.’ (Occupational Therapist, Trust two)

‘...does the way that it’s been done cause role blurring and some discomfort and confusion, yes it does because these things aren’t spelt out clearly... I think the lack of clarity it creates for social workers is for a number of reasons, one is that when we’ve done staff morale surveys, social workers tend to come out lowest and to feel the greatest degree of role blurring and a lowest sense of confidence and satisfaction in their work...’ (Consultant, Trust two)

‘I think having the separate psychology team helps keep professional boundaries in place, like having systems where we don’t become care co-ordinators in the same way and we don’t participate in assessments.’ (Psychologist, Trust two)

**Information systems:**

- **Information systems incompatibility**

**‘**We are still running dual systems and the difficulty is that no deal was done pre-integration to have a unified paperwork system in place.’ (Senior Manager, Nurse, Trust one)

‘We’ve actually got quite an old social services IT system which I think came out in the late 80s sometime … And it holds information which could be quite useful … if everybody could make use of it. But unfortunately the health service has got another system which doesn’t even have any remote likeness to our system. We’re running on two systems basically that don’t understand each other.’ (Team Leader, Social Worker, Trust two)

‘There are two computer systems and you cannot have software for both on one machine and that means that if you want to check whether someone is known to social services you’ve got to jump out of your seat and sit in that one and vice-versa. It’s a nuisance but it’s not insurmountable.’ (Team Leader, Social Worker, Trust one)

‘...Sometimes it’s extremely difficult with the amount of paperwork you’ve got to do for each patient and you can have patients that come and go every two days and you’re re-writing care plans and risk assessments. It can take a few days to get old notes of patients, so until they’re with us we don’t have a clue what their risk history is.’ (Ward CPN, Trust one)

- **IT provision**

‘I find it very difficult (not having a computer) because all of my training has been done via computer … I think the thing I’ve found most difficult is trying to manage paperwork without doing it directly onto a PC.’ (Social Worker, Trust one)

‘I have been trying to get a computer. There are three computers for a team of about fifteen people. It makes it difficult when you are asked to put on data electronically and you don’t have easy access to it, so it is difficult.’ (Psychologist, Trust two)

‘...we’ve got four machines between about five people and tempers really do fray.’ (Occupational Therapist, Trust two)

**Generic working**

‘People have huge case loads and been expected to take on other roles that they haven’t had the training for... don’t know where to go for information, for advice... something has to give... I think what we’re doing is becoming more and more diluted.’ (CPN, Trust two)

‘I think the social workers are particularly concerned … They’re being invited to do things like check on side effects of medication and that’s a problem.’ (Team Manager, Social Worker, Trust one)

‘I heard of another borough where they were advertising for a job and it was primarily a CPN’s role if you looked at it five years ago. And a social worker or an OT could apply for it and I thought, that is not good… And it’s dangerous because you don’t have continuity, you have a blurring of roles and people feel disempowered and leave and I think that’s what we’re having.’ (CPN, Trust two)

**Support for training and role development**

‘I can’t identify any training that any of us has been offered that’s accessible … We need stuff that’s not time consuming. We could definitely benefit from a lot more training from the social side. To recognize that our roles are changing.’ (Team Leader, CPN, Trust two)

‘We always have a problem that team members want to do training in other areas, social skills training, therapy training but then there is the issue of who is going to do the work while they are off.’ (Consultant, Trust one)

‘None of my CMHT leads have had management training.’ (Senior Manager, Nurse, Trust one)

**Workforce levels and workloads:**

- **Pressures on staffing levels**

‘Here they’ve cut back to save money. There are only twelve (in the team) whereas before there were a lot more of us. At least fifteen of us. So it is a small number for what we are supposed to do.’ (Social Worker, Trust one)

‘I think one of the biggest problems we have that we are under-resourced. All my staff have very high caseloads and it’s a struggle with the numbers of cases we have … I think I need two more social workers … and at least two more CPNs.’ (Team Leader, CPN, Trust two)

- **Recruitment, retention, staff sickness**

‘We had a very terrible time because one of our colleagues had a stroke, who is still recovering. The only CPN lady that we’ve got also became very unwell and was off for six months. So it means we’re relying on agency staff and I was the only permanent CPN … We haven’t had a consultant for over a year now. We had a locum consultant for, I think, four months and he left … I know they have been trying to recruit but they are finding it difficult to find the appropriate person to take up the position.’ (CPN, Trust one)

‘I’m aware that the CMHT that we relate to has a shortage of Registrars. The team is under-resourced.’ (GP, Trust one)

‘The three barriers to continuity of care are working in a difficult area where demand is impossibly high and that we ourselves can’t offer sufficient access of continuity to any client groups because we need GP recruitment, we need primary care team expansion to do that.’ (GP, Trust two)

- **Caseloads, caseload management**

‘The social workers are already stretched and the CPNs are already stretched as well who are carrying 35 plus cases. Even allocating people who are discharged from the ward is almost impossible because resources are stretched.’ (Team Leader, CPN, Trust one)

‘...each team normally has three CPNs. Because I’m the team leader, I have other responsibilities, that’s supposed to be half – the reality is, I’ve got a case load very similar to everybody else’s, but struggling to do the other part as well.’ (Team Leader, CPN, Trust two)

‘...a female CPN is leaving because of the workload...’ (Social Worker, Trust one)

‘ ...we have to maintain a certain caseload as a team and as individuals so therefore we are encouraged to have a throughput. Now obviously that can have an impact on people because you may have to move somebody from your caseload into another caseload or discharge them when perhaps they don’t feel they should be.’ (Psychologist, Trust one)

- **Administrative loads**

‘...patients and carers want to see in writing what they’re supposed to be getting. There are now Risk Assessment forms, Untoward Incidence forms, all these sorts of forms, so I don’t have data but I’m pretty sure that probably a proportion of CPN’s time is spent in paperwork now much more than it was five years ago, and no doubt they don’t welcome that.’ (Consultant Psychiatrist, Trust two)

‘We’ve got a very good secretary at the moment, but no matter how good they are, it’s impossible to do all that work, so what happens is that the secretary mostly just does doctors, outpatient matters. It’s not that doctors are more important than anyone else, it’s simply been which work needs to get out. So, nurses, social workers, OTs, have always written their own letters. We’re not experts at that and for me to do it will take an hour for what might take our secretary five, ten minutes to do.’ (Occupational Therapist, Trust two)

‘The paperwork shouldn’t interfere with (continuity) but it lengthens the initial phase spent in ‘now I’ve just got to get you to fill out this form.’ Some of it is useful... but the amount that we are being required to do is increasing so you have to collect more and more information before you even get down to ‘well, lets’ see what the issues are.’ (Psychologist, Trust two)

‘Over the last 15 or 16 years since I’ve worked in this area, you spent say 25 to 30% on paperwork and 70% on the coalface with your client. It’s now almost the reverse of that.’ (Social Worker, Trust one)

- **Impact of workloads on communication**

‘The relationships aren’t as good as they used to be because we don’t have the time to spend with each other like we used to.’ ( Voluntary Services Worker, Trust one)

‘More input, more communication. Sometimes it feels like there has to be a crisis before we get to talk.’ ( Voluntary Services Worker, Trust two)

**Service users’ needs:**

- **Complexity of needs**

‘I’ve felt this through the years that our clients have changed, we’re working with a lot more damaged people these days, with quite a long history of difficult behaviour, forensic history, quite a few people will carry on like that. People with drug and alcohol problems as well as mental illness. Dual diagnosis. And you know, the hostels have procedures that make it very difficult for these people to be accepted sometimes.’ (Social Worker, Trust one)

‘…over the last couple of years the client group has gotten younger and iller. More disturbed, more day hospital type. It feels like there’s been pressure to move people on from day hospitals. And we’re usually the next port of call…’ (Voluntary Sector Worker, Trust two)

‘…the biggest challenge I’ve seen coming up is around violence and substance misuse. They’re huge challenges that I think traditional mental health services are not equipped to deal with, and we need to be really focussing in on how we manage those things because they often help people, they’re often the issues that make people drop out of services.’ (Occupational Therapist, Trust two)

‘The second aspect is the large number of schizophrenic patients –we’d expect that in an inner city area, but what you wouldn’t expect is that a sizeable proportion, probably getting on for a third, just don’t have any contact with secondary care. It’s been the hidden group of psychotic patients that secondary care aren’t aware of.’ (GP Trust two)

- **Accommodation**

‘Well people end up going in to bed and breakfast accommodation which isn’t ideal and that’s very difficult to keep in contact with people in places like that.’ (Consultant Psychiatrist, Trust one).

‘Its (loss of day care) has had an impact on patients. ….It was a very convenient way of discharging people fairly safely that you could actually keep an eye on them….also preventing people from going into hospital.’ (Social Worker, Trust two).
